# Screening of medicinal plants for antibacterial principles against β-lactam resistant strains

**DOI:** 10.4103/0253-7613.75685

**Published:** 2011-02

**Authors:** N.V. Vinod, C. Sadasivan

**Affiliations:** Department of Biotechnology and Microbiology, Kannur University, Thalassery Campus, Palayad PO, Kannur - 670 661, Kerala, India

Sir,

In the present work, screening of different plants used in Indian folk medicine was carried out for the detection of antibacterial principles against β-lactam resistant bacterial strains, which may be useful as new drugs to combat deadly infections.[1,2] Twenty-nine plants were tested for their antibacterial activities. The collected plants were washed and shade dried. Twenty grams of the plant material was macerated and extracted with hexane, diethyl ether, methanol and water using a Soxhlet apparatus, based on their polarity. The dry fractions were made into a suspension using 10% dimethylsulfoxide (DMSO) in distilled water. The concentration of the extracts was adjusted to 1 mg/mL in each case. The antibacterial properties of the plant extracts were evaluated by employing the disc diffusion method. Evaluation of the synergistic effect of the plant extracts with β-lactam antibiotics was done according to the method of Muroi and Kubo.[3] The diethyl ether extracts of *Holoptelea integrifolia, Pongamea pinnata, Spondias dulci* and methanol extract of *Syzygium samarengense* showed good synergistic effects with amoxicillin and ampicillin against *Staphylococcus aureus* in disc diffusion method. They were further fractionated by silica gel chromatography to identify the antibacterial compound. The structure of the compounds in the active fractions was elucidated by spectroscopic analysis methods such as gas chromatography-mass spectrometry (GC-MS), Fourier Transform Infrared Spectroscopy (FTIR) and 1H nuclear magnetic resonance (NMR) analysis [Table 1]

**Table 1:**
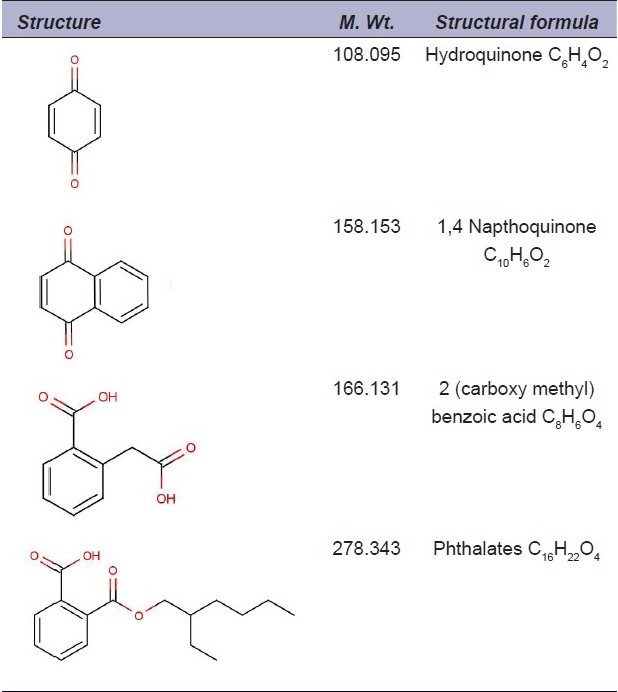
Some of the compounds identified from plant extracts

The diethyl ether extracts exhibited a higher degree of antimicrobial activity compared to methanol extract. The extracts of *H. integrifolia, P. pinnata, S. dulci* and *S. samarengense* showed an increase in the growth inhibition zone in the presence of amoxicillin. This indicates that the plants contain compounds which can enhance the effect of amoxicillin. Other plant extracts did not show any noticeable synergistic effect. The synergistic effect of plant extracts against resistant bacterial strains may lead to a new choice for the treatment of infectious diseases.[4] The active principle can be used along with commonly used antibiotics. Data from the literature as well as from our studies reveal a great potential of plants in this direction.

## References

[1] Fawcett CH, Spencer DM (1970). Plant chemotherapy with plant products. Ann Rev Phytopath.

[2] Sharma KK, Sangrraula H, Mediratta PK (2002). Some new concepts in antibacterial drug therapy. Indian J Pharmacol.

[3] Muroi H, Kubo I (1996). Antibacterial activity of anacardic acids and totarol alone and in combination with methicillin, against methicillin-resistant *Staphylococcus aureus*. J Appl Bacteriol.

[4] Nacimento GG, locatelli J, Freitas PC, Silva GL (2000). Antibacterial activity of plant extracts and phytochemicals on antibiotic-resistant bacteria. Braz J Microbiol.

